# Do women with high-risk HPV E6/E7 mRNA test positivity and NILM cytology need colposcopy?

**DOI:** 10.1186/s13027-023-00531-w

**Published:** 2023-09-29

**Authors:** Ying Liu, Xiu Jin, Yingying Gong, Yingying Ma, Beibei Du, Linqing Yang, Yunfei Wang, Weipei Zhu

**Affiliations:** 1 Department of Obstetrics and Gynecology, The Second Affiliated Hospital of Soochow University, Suzhou, 215000 China; 2 Department of Gynecology, Affiliated Hospital of Jining Medical University, Shandong, 272000 China

**Keywords:** Cervical screening, HPV E6/E7 mRNA, NILM, Genotyping

## Abstract

**Purpose:**

This study aimed to assess the value of an HPV E6/E7 mRNA assay and HPV 16 18/45 genotype assay combined with age stratification for triaging women negative for intraepithelial lesions or malignancy (NILM) cytology.

**Methods:**

From January 2017 to December 2021, a total of 162,309 eligible women underwent cervical cancer screening at the Affiliated Hospital of Jining Medical University, China. Excluding those with negative HPV E6/E7 mRNA, abnormal and unsatisfactory cytology, and those who failed to undergo colposcopy, 6,845 women were ultimately included in our study. We analysed the triage guidance for different subtypes of HPV in the presence of NILM cytology.

**Results:**

Among 162,309 women, 19,834 (12.2%) were positive for HPV E6/E7 mRNA. Of the 6,845 women included in the study, 1,941 (28.4%), 561 (8.2%), 55 (0.8%) and 4,288 (62.6%) tested positive for HPV 16, HPV 18/45, HPV16/18/45 or other HR-HPV genotypes, respectively. The proportions of LSIL+ (including LSIL, HSIL and ICC) and HSIL+ (including HSIL and ICC) pathological results in the HPV 16/18/45 + group were 57% and 34.1%, respectively, higher than 36.3% and 11% in the other HR-HPV + group (χ^2^ = 653.214, P < 0.001). The percentages of LSIL + and HSIL + in the HPV16 + group (61.3% and 42.8%, respectively) and HPV16+/18/45 + group (76.3% and 41.9%, respectively) were much higher than those in the HPV18 + group (40.6% and 13.1%, respectively) (P < 0.001). However, there was no significant difference in the percentage of histopathological results between the HPV16 + group and HPV16+/18/45 + groups (P > 0.05). The above results were consistent after stratification according to age.

**Conclusion:**

The rate of histopathological abnormalities was still high for the other HR-HPV subtypes with NILM cytology, although the rate of histopathological abnormalities was much higher for the HPV 16/18/45 positive subtypes. Therefore, colposcopy should be performed in women with HPV E6/E7 mRNA positivity and NILM cytology, regardless of age and HPV genotype.

## Introduction

Cervical cancer is a serious cancer that threatens women’s health. Cervical cancer incidence and mortality have increased yearly in China over the last 20 years, and the impact on young women has also increased [1–3]. The main cause of cervical cancer is persistent infection with high-risk human papillomavirus (HPV) [4, 5]. Cervical cancer prevention is universal around the world. However, cervical cancer vaccination began late in China and vaccination coverage is low [6, 7]. As a result, it is critical that we upgrade our current screening mechanism.

Traditional Pap screenings have performed poorly in underdeveloped countries, with a sensitivity ranging from 30 to 40% [8]. Despite the improved performance of liquid-based cytology, the number of cytopathologists in the country remains inadequate, and diagnostic skills vary, limiting the use of this technology in routine screening. Zhao et al. [9] reported that liquid-based cytology had lower sensitivity for detecting CIN2+ (80.7%). Given the oncogenic cause, HPV testing can be a valid technique for diagnosing the risk of cervical cancer in women. Women who have normal sexual intercourse have a lifetime possibility of being infected with at least one type of HPV of up to 80% [10], but most HPV infections are transient; because HPV DNA testing cannot identify transient HPV infections, it leads to unnecessary follow-up and even overtreatment of HPV-infected patients, increasing the financial and psychological burden of patients [11].

The E6/E7 oncogenes are well known to play an important role in the development of cervical cancer. Because E6/E7 overexpression occurs after HPV integration into the genome, direct detection of HR-HPV E6/E7 in cervical samples may be more specific in detecting high-grade cervical lesions than HR-HPV-DNA testing [12]. Compared with HPV DNA testing using a noninferiority scoring method, HPV E6/E7 mRNA testing passed the cross-sectional clinical and reproducibility requirements of international CIN2 + detection HPV testing [13, 14]. As a result, the number of patients who use E6/E7 mRNA for HPV testing is increasing yearly in China [15–17]; however, there is a lack of uniform clinical standards and guidelines for the management of HPV E6/E7 mRNA-positive patients, and an increasing number of patients who are positive for genotypes other than 16 and 18/45 are being referred for colposcopy and cervical biopsy. As a result, a major study evaluating triage techniques for different genotypes of HPV E6/E7 mRNA positivity in women with normal liquid-based cytology is needed.

In this study, we analysed the pathological diagnosis of cervical biopsy in patients positive for different genotypes of HPV E6/E7 mRNA in the presence of normal liquid-based cytology. We preliminarily discussed the need for referral colposcopy and cervical biopsy in patients positive for different genotypes of HPV E6/E7 mRNA to provide feasible suggestions and a basis for clinicians for follow-up management.

## Methods

### Study population and study design

**Fig. 1:**
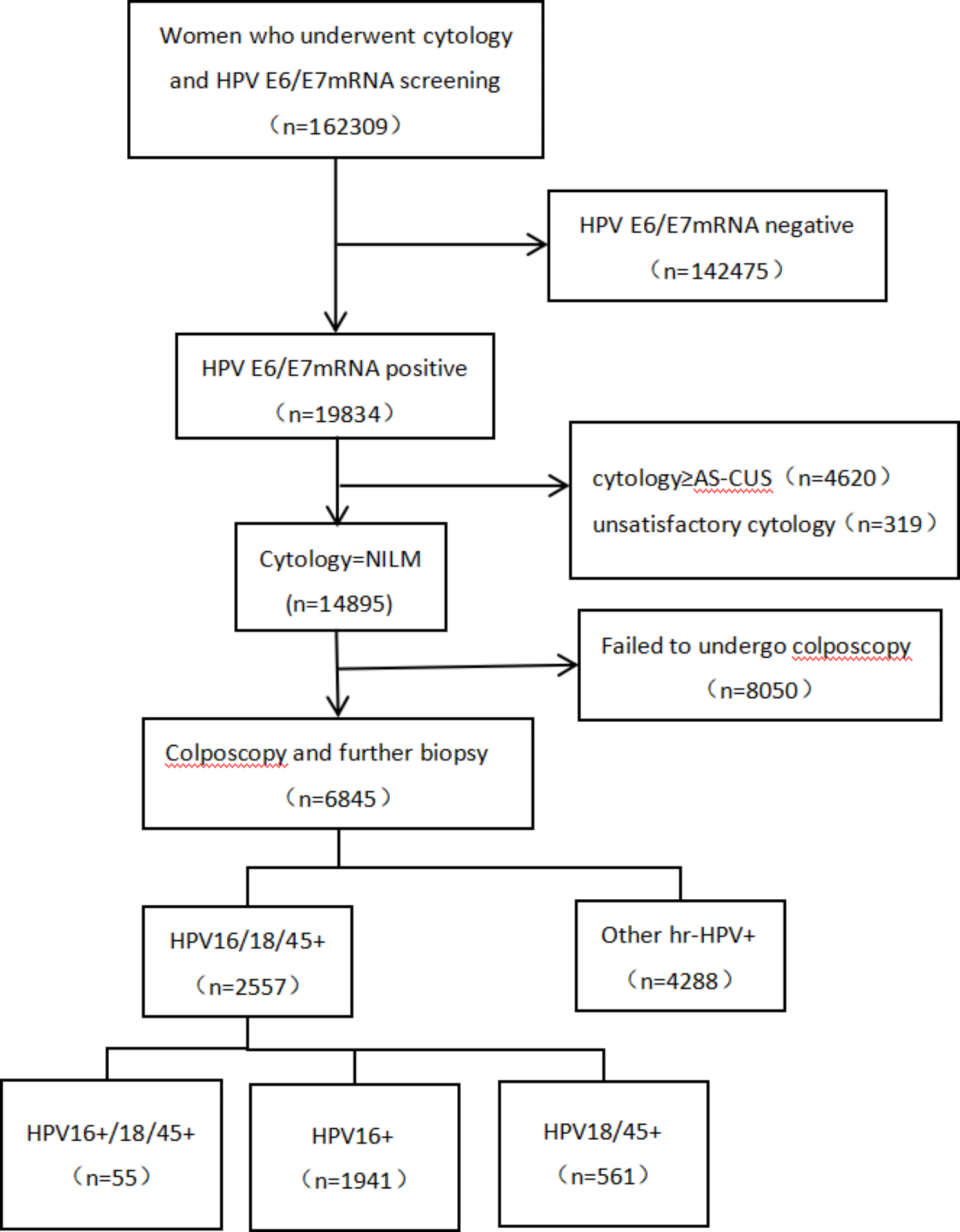
Management procedure, the results and outcomes. ASC-US, atypical squamous cells of undetermined significance; NILM, negative for intraepithelial lesions or malignancies; HPV, human papillomavirus; HPV16/18/45+, HPV16 + or HPV18/45+; HPV16+, HPV16 + and HPV18/45-; HPV18/45+, HPV16- and HPV18/45+; HPV16+/18/45+, HPV16 + and HPV 18/45+

From January 2017 to December 2021, a total of 162,309 eligible women aged 18–75 years underwent cervical cancer screening in the physical examination centre and gynaecological clinic of the Affiliated Hospital of Jining Medical University, China. Women were excluded from the study according to the following criteria: (1) previously confirmed cervical cancer or other malignancies; (2) pregnancy; or (3) unsatisfactory cytology. A total of 19,834 HPV E6/E7 mRNA-positive women were detected among the 162,309 women. A total of 4939 women were excluded because of abnormal liquid-based cytology (LBC) and unsatisfactory cytology. Because colposcopy and cervical biopsies were not conducted in 8050 women, they were excluded from the study. Therefore, valid data for all tests were available for 6845 women, as shown in Fig. 1.

### Liquid-based cytology

LBC technology was used for cytological detection. Thin-layer slides were created using the Thin Prep 2000 Processor (Cytyc Corporation, Marlborough, MA, USA) according to the manufacturer’s instructions. Cytologic data were simultaneously and independently diagnosed by two expert cytopathologists without knowledge of the other examination results. The results of LBC were evaluated according to the Bethesda system 2014.

### HPV E6/E7 mRNA testing

HR-HPV E6/E7 mRNA was detected using the Aptima® HPV assay (Gen-Probe; Hologic, San Diego, CA), an FDA-certified HPV E6/E7 mRNA assay that detects 14 h-HPV types (HPV16, 18, 31, 33, 35, 39, 45, 51, 52, 56, 58, 59, 66, and 68), according to the manufacturer’s instructions. All HR-HPV-positive samples were further genotyped by the Aptima® HPV 16 18/45 Genotype (GT) assay (AHPV-GT) (Gen-Probe; Hologic, San Diego, CA, USA). AHPV-GT can detect HPV16 and a subset of HPV18 and HPV45 cases [17].

### Colposcopy and histological diagnosis

A senior colposcopist (5 years of experience or more) performed the colposcopy utilising an electronic colposcopy system (Kinkoway, Shenzhen, China), and the results were reviewed by another senior colposcopist (5 years of experience or more). The cervical transformation zone (normal, low-grade lesion (LGL) and high-grade lesion (HGL)) was evaluated using standard colposcopic procedures according to the International Federation of Cervical Pathology and Colposcopy (IFCPC) 2011 criteria. A biopsy was taken at the most severe lesion in the four-quadrant transformation zone of the cervix for those with suspicious or abnormal colposcopy; if no abnormality was seen, a random biopsy was performed in the four-quadrant transformation zone of the cervix. If the transformation zone was not completely visible, an endocervical curettage (ECC) was performed.

Two experienced pathologists independently diagnosed histological slides without knowing the results of the other assays. If the two pathologists gave different diagnoses, then a discussion was held by all pathologists in our hospital until a consensus was reached. The diagnosis result of histological slides meets the standard of the current World Health Organisation classification. The pathological classification included the following: (1) normal or chronic inflammatory changes were classified into the normal group; (2) CIN1- and p16-negative CIN2 were classified into the LSIL group; (3) p16-positive CIN2, CIN3 and cervical carcinoma in situ were classified into the HSIL group; and (4) invasive squamous cell carcinoma and adenocarcinoma were classified into the ICC group [18].

### Statistics

SPSS Statistics 21.0 (IBM Corp., Armonk, New York, USA) was used for statistical analysis. A P value < 0.05 (two-sided) was considered statistically significant. Pearson’s chi-square or Fisher’s exact probability test was used to compare the differences in percentages between different groups. The 95% confidence intervals (CIs) of proportions were calculated based on the following equation: $p\pm 1.96\sqrt{p\left(1-p\right)/n}$, where n is the number of cases involved.

## Results

### Percentage of different HPV genotypes

As shown in Fig. 1, of the 6845 HPV E6/E7 mRNA-positive women, a total of 4288 (62.6%) were other hr-HPV (31, 33, 35, 39, 51, 52, 56, 58, 59, 66, and 68) positive and 2557 (37.4%) were HPV16/18/45 positive, of which 1941 (28.4%) were HPV16 positive, 561 (8.2%) were HPV18/45 positive, and 55 (0.8%) were both HPV16 and HPV18/45 positive.

### Age distribution

**Fig. 2:**
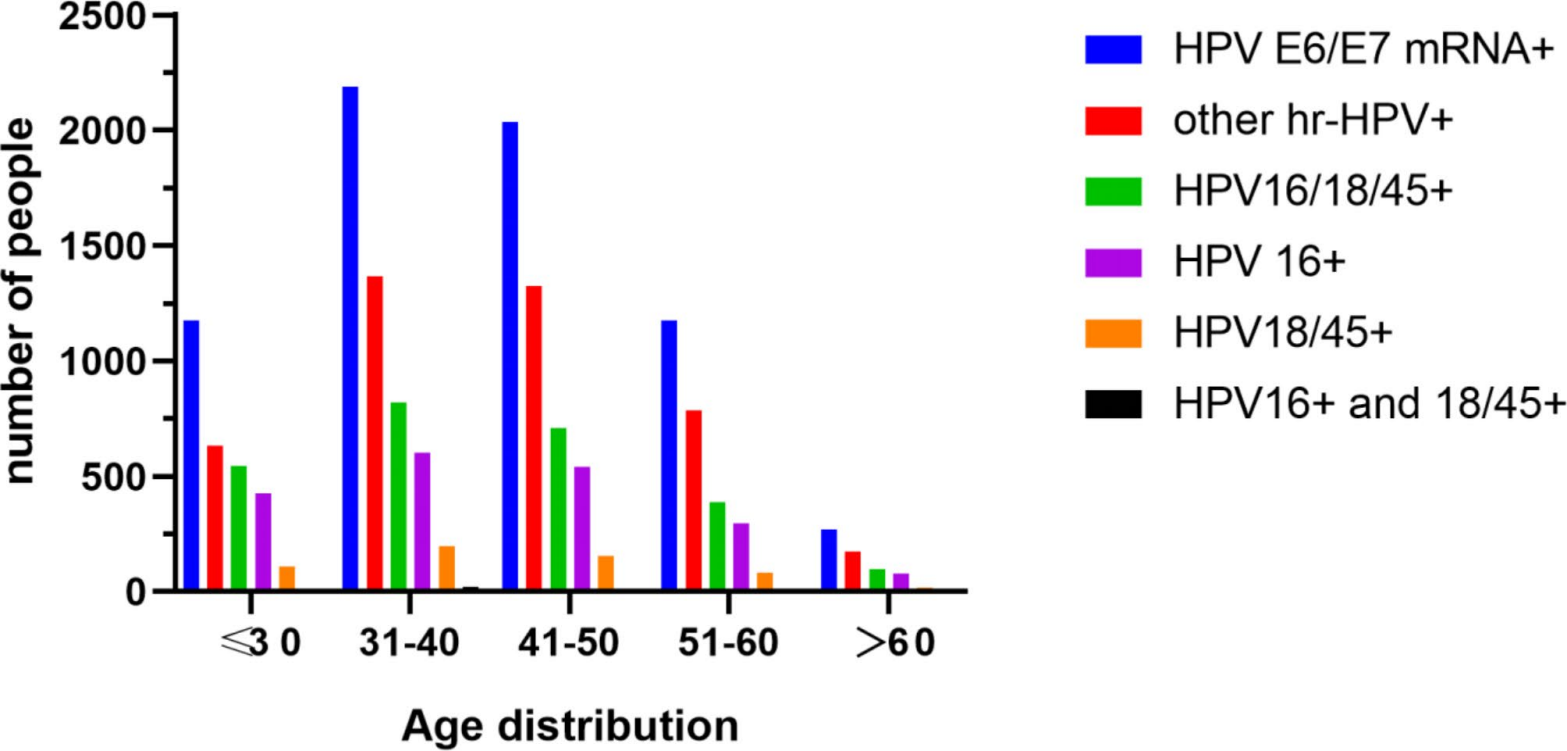
The age distribution of 6845HPV E6/E7 mRNA-positive women

The median age of the 6845 women recruited was 41.42 ± 10.69 years. We calculated the age distribution of all enrolled cases. As shown in Table 1; Fig. 2, of the 6845 HPV E6/E7 mRNA-positive women, a total of 2189 (32.0%, 95% CI: 30.9–33.1%) were aged 31–40 years, which was the highest number among all groups. Similarly, the proportion of people aged 31–40 years was the highest for each HPV genotype, while the proportion of people aged > 60 years was the lowest.

**Table 1:** Age Distribution of 6845 women with HPV E6/E7 mRNA test positivity and NILM cytology

	≤ 30years	31-40years	41-50years	51-60years	>60years
Number E6/E7mRNA+	1176	2189	2035	1175	270
(% of age group; 95% CI)	17.2%(16.3–18.1%)	32.0%(3.1–3.3%)	29.7%(28.6–30.8%)	17.2%(16.3–18.1%)	3.94%(3.5–4.4%)
Number Other-hrHPV+	633	1369	1326	786	174
(% of age group; 95% CI)	9.25%(8.6–9.9%)	20.0%(19.1–20.9%)	19.4%(18.4–20.3%)	11.5%(10.7–12.2%)	2.5%(2.2–2.9%)
Number HPV16/18/45+	543	820	709	389	96
(% of age group; 95% CI)	7.9%(7.3–8.6%)	12.0%(11.2–12.7%)	10.4%(9.6–11.1%)	5.7%(5.1–6.2%)	1.4%(1.1–1.7%)
Number HPV16+	425	603	540	297	77
(% of age group; 95% CI)	6.2%(5.6–6.8%)	8.8%(8.1–9.5%)	7.9%(7.3–8.5%)	4.3%(3.9–4.8%)	1.1%(0.9–1.4%)
Number HPV18/45+	109	197	155	82	18
(% of age group; 95% CI)	1.6%(1.3–1.9%)	2.9%(2.5–3.3%)	2.3%(1.9–2.6%)	1.2%(0.9–1.5%)	0.3%(0.1–0.4%)
Number HPV16+/18/45+	9	20	14	10	1
(% of age group; 95% CI)	0.1%(0.0-0.2%)	0.3%(0.2–0.4%)	0.2%(0.1–0.3%)	0.1%(0.1–0.2%)	0.0%(0.0–0.0%)

HPV16/18/45+:HPV16 + or HPV18/45+; HPV16+:HPV16 + and HPV18/45-; HPV18/45+:HPV16- and HPV18/45+; HPV16+/18/45+:HPV 16 + and HPV 18/45+

### Histopathological results for different HPV subgenotypes

As shown in Table 2, we compared the histopathological results of the HPV16/18/45 + group to those of the other HR-HPV + group and discovered that 43.0% of the HPV16/18/45 + group had normal histopathological results, which was significantly lower than the proportion of the other HR-HPV-positive group with normal histology (65.9%). In contrast, the proportions of HSIL and ICC pathological results in the HPV 16/18/45 + group were 28.4% and 7.9%, respectively, higher than 8.9% and 2.1% in the other HR-HPV + group. (χ2 = 653.214, P < 0.001). However, the percentage of LSIL was not significantly different between the two groups (20.7% and 23.3%, respectively). We divided the 2557 patients in the HPV16/18/45 + group into HPV16+, HPV18/45 + and HPV16+/18/45 + groups and compared the histopathological results as shown in Table 3. The percentages of HSIL + in the HPV16 + group (42.8%) and HPV16+/18/45 + group (41.9%) were much higher than that in the HPV18/45 + group (13.1%) (P < 0.001). Interestingly, there was no significant difference in the percentage of histopathological results between the HPV16 + group and HPV16+/18/45 + groups (P > 0.05).

**Table 2:** Histopathological results in the HPV16/18/45 + group and the other HR-HPV + group.

		HPV 16/18/45 +,n(%)	Other HR-HPV+,n(%)	χ^2^ value	P value
Histology results	Normal	1100(43.0)^a^	2824(65.9)^b^	653.214	< 0.001
	LSIL	529(20.7)^a^	993(23.2)^a^		
	HSIL	726(28.4)^a^	380(8.9)^b^		
	ICC	202(7.9)^a^	91(2.1)^b^		

Comparing the same line, there is a difference between a and b (P<0.05)

HPV16/18/45+, HPV16 + or HPV18/45+; HR-HPV, high-risk HPV; LSIL, low-grade squamous intraepithelial lesion; HSIL, high-grade squamous intraepithelial lesion; ICC, invasive cervical cancer

**Table 3:** Histopathological results in the HPV16+/18/45 + group, HPV16 + group and HPV18/45 + group.

		HPV16+/18/45+,n(%)^a^	HPV 16+,n(%)^a^	HPV18/45+,n(%)^b^	χ^2^ value	P value
Histology results	Normal	16(23.7)	751(38.7)	333(59.4)		< 0.001
	LSIL	16(29.1)	359(18.5)	154(27.5)		
	HSIL	20(36.4)	662(34.1)	44(7.8)		
	ICC	3(5.5)	169(8.7)	30(5.3)		

Statistically significant difference between a and b (P < 0.001)

HPV16+: HPV16 + and HPV18/45-; HPV18/45+: HPV16- and HPV18/45+; HPV16+/18/45+: HPV 16 + and HPV 18/45+; LSIL: low-grade squamous intraepithelial lesion; HSIL: high-grade squamous intraepithelial lesion; ICC: invasive cervical cancer

### Histopathological results of different HPV subgenotypes after stratifying by age

To determine the impact of HPV E6/E7 mRNA at different ages, we stratified the analysis by age ≤ 40 and > 40 years and found that the proportion of LSIL+ (including LSIL, HSIL and ICC) was higher in the HPV16/18/45 + group than in the other HR-HPV + group, regardless of age ≤ 40 or > 40 years (χ2 = 307.735, P < 0.001 and χ2 = 359.11, P < 0.001). We also compared the pathological findings in the HPV16+, HPV18/45 + and HPV16+/18/45 + groups in both age groups. In either age group, the proportion of LSIL + cells in the HPV16 + and HPV16+/18/45 + groups was higher than that in the HPV18 + group (χ2 = 103.829, P < 0.001 and χ2 = 80.497, P < 0.001), while there was no significant difference between the HPV16 + group and HPV16+/18/45 + group. See Tables 4 and 5 for details.

**Table 4:** Histopathological results in the HPV16/18/45 + group and the other HR-HPV + group after stratifying by age

		Age ≤ 40		Age > 40	
		HPV 16/18/45 +, n(%)	Other HR-HPV+, n(%)	HPV16/18/45, n+(%)	Other HR-HPV+, n(%)
Histology results	Normal	595(43.7)	1301(65.0)	505(42.3)	1523(66.6)
	LSIL	314(23.1)	510(25.5)	215(18.0)	483(21.1)
	HSIL	406(29.8)	179(8.9)	320(26.8)	201(8.8)
	ICC	47(3.5)	12(0.6)	155(13.0)	79(3.5)
χ^2^ value		307.735		359.11	
P value		< 0.001		< 0.001	

HPV16/18/45+: HPV16 + or HPV18/45+; HR-HPV: high-risk HPV; LSIL: low-grade squamous intraepithelial lesion; HSIL: high-grade squamous intraepithelial lesion; ICC: invasive cervical cancer

**Table 5:** Histopathological results in the HPV16+/18/45 + group, HPV16 + group and HPV18/45 + group after stratifying by age

		Age ≤ 40			Age > 40		
		HPV16+/18/45+,n(%)^a^	HPV16+, n(%)^a^	HPV18/45+,n(%)^b^	HPV16+/18/45+,n(%)^a^	HPV16+, n(%)^a^	HPV18/45+,n(%)^b^
Histology results	Normal	10(34.5)	404(39.3)	181(59.2)	6(23.1)	347(38.0)	152(59.6)
	LSIL	7(24.1)	212(20.6)	95(31.0)	9(34.6)	147(16.1)	59(23.1)
	HSIL	10(34.5)	375(36.5)	21(6.9)	10(38.5)	287(31.4)	23(9.0)
	ICC	2(6.9)	36(3.5)	9(2.9)	1(3.8)	133(14.6)	21(8.2)
χ^2^ value		103.829			80.497		
P value		< 0.001			< 0.001		

Statistically significant difference between a and b (P < 0.001)

HPV16+:HPV16 + and HPV18/45-; HPV18/45+: HPV16- and HPV18/45+; HPV16+/18/45+: HPV 16 + and HPV 18/45+; LSIL: low-grade squamous intraepithelial lesion; HSIL: high-grade squamous intraepithelial lesion; ICC: invasive cervical cancer

## Discussion

As E6/E7 mRNA is only expressed in actively infected cells, total transcript levels increase during CIN occurrence and progression. The E6/E7 mRNA-based HPV test was found to be more specific in detecting high-grade lesions than the DNA-based HPV test [19, 20]. Therefore, the HPV E6/E7 mRNA test is more suitable for primary screening of cervical cancer. However, no previous report has presented a strategy to manage HPV E6/E7 mRNA-positive women, especially those with NILM cytology.

Previous large population-based studies in China reported that the HPV infection rate was 9.9-27.5% [21]. In this study, the prevalence of HPV infection was 12.2%, which was at the low end of the reported range of HPV prevalence in China. The possible reason for this is that we tested for HPV E6/E7 mRNA, not HPV DNA, and the positive rate may be low. Previous studies have also found lower positive rates for HPV E6/E7 mRNA testing than for HPV-DNA testing [22]. Another two reasons for different rates: this study excluded the women showing abnormal cytology; differences in the age distribution of women because the HPV prevalence is higher in younger women.HPV 16, 18 and 45 are more likely to be integrated into the human genome than other HPV types [23] and account for approximately 76% of invasive cervical cancers worldwide [24]. The proportion of HPV16 in invasive cervical cancer in Asia was 60.5%, which was significantly lower than the rate of 72% in North America [24]. Compared with 8.5% in the CLEAR study [25] and 8.2% in the ATHENA study [26, 27], the positive rate of HPV16 in women with NILM cytology in our study was lower (3.5%), which was mainly due to different subtypes of HPV infection that vary by race and region.

The use of the AHPV-GT test, which can detect HPV E6/E7 mRNA in HR-HPV genotypes 16 and 18/45, to triage women with NILM cytology has been reported in very few studies. In this study, the immediate risk of HISL+ (including HSIL and ICC) in women with HPV16/18/45-positive NILM cytology was significantly higher than that in other HR-HPV-positive women (36.3% vs. 11%, P < 0.001). However, the immediate risk of HSIL + remains high in other HR-HPV-positive women, and this result does not change with age stratification. The 2019 ASCCP guideline recommends colposcopy when immediate CIN3 + risk is 4–24%. [28, 29]. The above results support immediate referral for colposcopy in women with HPV E6/E7 mRNA-positive NILM cytology, regardless of age and HPV genotype.

Meanwhile, we grouped the three HPV16/18/45 subtypes again and found that the detection rates of HSIL and SCC were significantly higher in the HPV16 subtype-positive group than in the HPV18/45 subtype-positive group (42.8% vs. 13.1%, P < 0.001), whereas the detection rates of HSIL and SCC did not increase when HPV16 was combined with HPV18/45 infection compared with single HPV16 infection (42.8% vs. 41.9%, P>0.05). Similarly, the results did not change with age stratification. We should therefore be more alert for patients with positive HPV16 subtypes.

This study has several limitations. First, because our data were derived from a clinic-based population rather than a general population, biases may exist. Second, this study did not exclude patients with a previous diagnosis of CIN, and there may be a bias, such as a higher proportion of histological LISL+ (42.7%) in HPV E6/E7 mRNA-positive patients. Third, the analysis of this study was limited to baseline data and did not include follow-up surveillance data; therefore, our study does not represent a conventional screening program and it may be more suitable for countries that have no active screening program. What’s more, follow-up studies of women with positive HPV E6/E7 mRNA and NILM cytology are needed to further verify the conclusions of this study.

## Conclusion

In summary, this study found that the rate of histopathological abnormalities remained high for the other HR-HPV subtypes with NILM cytology, but the rate of histopathological abnormalities was much higher for HPV 16/18/45 subtype positivity. Therefore, in clinical work-up, colposcopy should be performed in women with HPV E6/E7 mRNA-positive NILM cytology, regardless of age and HPV genotype. There should be more vigilance for HPV 16 subtype infection.

## List of abbreviations

ASC-US: Atypical squamous cells of undetermined significance;
NILM: Negative for intraepithelial lesions or malignancies;
HPV: Human papillomavirus;
HR-HPV: High-risk HPV;
HPV16/18/45+: HPV16 + or HPV18/45+;
HPV16+: HPV16 + and HPV18/45-;
HPV18/45+: HPV16- and HPV18/45+;
HPV16+/18/45+: HPV16 + and HPV 18/45+;
LSIL: Low-grade squamous intraepithelial lesion;
HSIL: High-grade squamous intraepithelial lesion;
ICC: Invasive cervical cancer
