# Correction: Do women with high-risk HPV E6/E7 mRNA test positivity and NILM cytology need colposcopy?

**DOI:** 10.1186/s13027-023-00554-3

**Published:** 2023-12-11

**Authors:** Ying Liu, Xiu Jin, Yingying Gong, Yingying Ma, Beibei Du, Linqing Yang, Yunfei Wang, Weipei Zhu

**Affiliations:** 1 Department of Obstetrics and Gynecology, The Second Affiliated Hospital of Soochow University, Suzhou, 215000 China; 2 Department of Gynecology, Affiliated Hospital of Jining Medical University, Shandong, 272000 China

**Infectious Agents and Cancer (2023) 18:54**

10.1186/s13027-023-00531-w

After publication of this article [1], the authors reported that in this article all authors were assigned to affiliations 1 and 2, but it should be as follows:

Ying Liu: 1 and 2;

Xiu Jin, Yingying Gong, Yingying Ma, Beibei Du, Linqing Yang and Yunfei Wang: 2;

Weipei Zhu: 1.

The original article [1] has been corrected.
